# 

**Published:** 1836-10

**Authors:** 


PAGE

Art. I.—Traité Clinique des Maladies du Cœur, précédé de Recherches nouvelles sur l'Anatomie et la Physiologie de cet Organe. Par J. Bouillaud, Professeur de Clinique Médicale à la Faculté de Médecine de Paris . . 309
A Clinical Treatise on the Diseases of the Heart, preceded by original Researches on the Anatomy and Physiology of that Organ. By J. Bouillaud, Clinical Professor to the Faculty of Medicine at Paris.

Art. II.—A Treatise on Diseases of the Eye and its Appendages. By Richard Middlemore, m.r.c.s., Surgeon to the Birmingham Eye Infirmary . 342

Art. III.—Analekten ueber Kinderkrankheiten: oder Sammlung ausserwählter abhandlungen ueber die krankheiten des kindlichen alters, zusammengestellt zum gebrauche für praktische Aerzte. Heft I.—VIII. . . 353
Selections in Children's Diseases; or, a Collection of Select Treatises on Infantile Diseases, for the use of practical Physicians. Parts I.—VIII.

Art. IV.—The Physiology of Digestion, considered with Relation to the Principles of Dietetics. By Andrew Combe, m.d. . . . 368

Art. V.—1. Médecine Légale, Théorique et Pratique, par Alph. Devergie, Docteur en Médecine, Professeur agrégé de la Faculté de Médecine de Paris, Professeur de Médecine Légale et de Chimie Médicale, &c. Avec le Texte, et l'Interprétation des Lois relatives à la Médecine Légale, revus et annotés par J. B. F. Dehaussy De Robecourt, Conseiller à la Cour de Cassation, &c. Tom. I., Tom. II. P. I. . . . . . 390
Forensic Medicine, Theoretical and Practical, by Alph. Devergie, &c. With the Text, and Explanations of the Laws relating to Forensic Medicine, reviewed and commented upon by J. B. F. Dehaussy Robecourt.

2. Elements of Medical Jurisprudence. By Alfred S. Taylor, f.l.s ,Lecturer on Medical Jurisprudence and Chemistry in Guy's Hospital. Vol. I. . . 390

Art. VI.—1. Nouvelles Recherches sur la Structure de la Peau. Par M. G. Breschet et M. Roussel de Vauzeme . . . . 429
New Enquiries on the Structure of the Skin. By M. G. Breschet and M. Roussel de Vauzeme.

2. Vergleichende Untersuchungen über die Haut des Menschen und des Haus-Saügethiere, besonders in Beziehung auf die Absonderungsorgane des Haut-Talges und des Schweisses. Von Gurlt . . . . ib.
On the Comparative Anatomy of the Skin of Man and the Domestic Mammalia, more particularly as it regards the Organs of the Oleaginous Secretion and of the Perspiration. By Professor Gurlt.

3. Ueber die menschliche Epidermis. Von Dr. Alphons Wendt . . ib.
On the Human Epidermis. By Dr. Alphons Wendt.

Art. VII.—Illustrations of the Elementary Forms of Disease. By Robert Carswell, m.d., Professor of Pathological Anatomy in the University of London, &c. Fasciculus IX. and X. . . . . 456

Art. VIII.—Rapport et Discussions à l'Académie Royale de Médecine sur la Taille et la Lithotritie, &c. . . . . . . 466

Report and Discussion at the Royal Academy of Medicine, on Lithotomy and Lithotrity, &c.

### PART SECOND.—Bibliographical Notices.

PAGE

ART. I.—Lectures on Subjects connected with Clinical Medicine. By P. M. LATHAM, M.D., Fellow of the Royal College of Physicians, and Physician to St. Bartholomew's Hospital . . . . . . 473

ART. II.—An Enquiry into the Pathology, Causes, and Treatment of Puerperal Fever: being an Essay for which the Fothergillian Gold Medal was conferred on the Author, by the Medical Society of London, in March, 1835. By G. MOORE, F.R.C.S. &c. . . . . . . 481

ART. III.—Medical Commentaries on Puerperal Fever, Vermination, and Water in the Head. By JOHN ALEXANDER, M.D., Medical Officer to the General Dispensary for Children, and Bank of Manchester, &c. . . . 490

ART. IV.—An Essay on the Operation for Cleft Palate. By GEORGE BUSHE . 493

ART. V.—On the Medical Properties of the Natural Order Ranunculaceæ; and more particularly on the uses of Sabadilla Seeds, Delphinium staphisagria, and Aconitum napellus, and their Alcaloids, Veratria, Sabadilline, Delphinia, and Aconitine. By A. TURNBULL, M.D. . . . . 499

ART. VI.—1. Ueber die Augenkrankheit, welche in der Belgischen Armee herrscht. Nebst einigen Bemerkungen über die Augenkrankheiten am Rheine, und über Augenblennorrhoeen im Allgemeinen. Von J. C. JÜNGKEN, u. s. w. . 501

On the Ophthalmia which prevails in the Belgian Army: with some Remarks on the Eye-Diseases on the Rhine, and Purulent Ophthalmia in general. By J. C. JÜNGKEN, M.D., Prof. of Medicine in the University of Berlin, &c.

2. Ueber die Augenkrankheit welche in der Kaiserlich-Russischen activen Armee herrscht. Von ROMAN TSCHETIRKIN, M.D. &c. Aus dem Russischen, von Dr. MAGAZINER . . . . . . . ib.

On the Ophthalmia prevalent in the Imperial Russian Army. By Dr. TSCHETIRKIN. Translated from the Russian by Dr. MAGAZINER.

ART. VII.—A Dictionary of Terms employed by the French, in Anatomy, Physiology, Pathology, Practical Medicine, Surgery, Midwifery, &c. &c.; with their Derivation from the Greek and Latin; their Synonyms in the Greek, Latin, French, German, and English; and Illustrations in the different Languages. By SHIRLEY PALMER, M.D. Parts I. and II. (A.—Hui.) . 508

ART. VIII.—Traité Théorique et Pratique sur les Altérations organiques simple et cancéreuses de la Matrice. Par F. DUPARCQUE, D.M. &c. . . 511

A Theoretical and Practical Treatise upon simple and cancerous Organic Alterations of the Uterus, &c.

ART. IX.—Medical and Physical Researches; or, Original Memoirs in Medicine, Surgery, Physiology, Geology, Zoology, and Comparative Anatomy. Illustrated with Plates, containing 160 Figures. By R. HARLAN, M.D. F.L.S. London; Surgeon to the Philadelphia Almshouse Hospital; Professor of Comparative Anatomy, &c. . . . . . . 518

ART. X.—Einige Bemerkungen über den einfluss der Witterung auf den Menschlichen organismus überhaupt, und insbesondere auf die anwendung der Seebäder in Dobberan. Vom Dr. JOHANN H. BECKER . . . 520

Remarks on the Influence of the Weather on Man, and on the Use of Sea-bathing. By J. H. BECKER, M.D.

ART. XI.—The Obstetrician's Vade Mecum; or, Aphorisms on Natural and Difficult Parturition; the application and use of Instruments in Preternatural Labours, or Labours complicated with Hemorrhage, Convulsions, &c. By THOMAS DENMAN, M.D. &c. Considerably augmented, and arranged according to the present State of Obstetricy, by MICHAEL RYAN, M.D. Ninth Edition. Illustrated with seventeen Plates, and a Portrait of the Author . . 523

PAGE

ART. XII.—Diagnostisch-praktische Abhandlungen aus dem Gebiete der Medicin und Chirurgie durch Krankheitsfälle erläutert. Vom Dr. LÖWENHARDT. Erster Theil . . . . . . 526
Diagnostic and Practical Essays on Medical and Surgical Subjects, illustrated by Cases. By Dr. LÖWENHARDT. Part First.

ART. XIII.—Erfahrungen ueber die Anwendung der Kälte in Krankheiten. Von J. D. BRANDIS, M.D. Königl. Dänischen Leibarzte, &c. . . . 529
Practical Observations on the Use of Cold in the Treatment of Disease. By J. D. BRANDIS, M.D. Physician in Ordinary to the King of Denmark, &c.

ART. XIV.—Della artificiale Riduzione a Solidità Lapidea e Inalterabilita degli Animali, scoperta da GIROLAMO SEGATO. Relazione dell' Avocato GIUSEPPE PELLEGRINI . . . . . . 532
Of the artificial Conversion of Animals to a State of stony Induration and Indestructiveness, discovered by GIROLAMO SEGATO. By G. PELLEGRINI.

---

### PART THIRD.—Selections from Foreign Journals.

### MORBID ANATOMY.

Case of Aneurism of the Thoracic Duct. By Dr. ALBERS, of Bonn . 535

On the Anatomical Condition of Diseased Bone. By Professor GERDY . ib.

### PHYSIOLOGY.

On the Physiology of Vomiting; and on the Causes of its Difference in Adults and Children. By Professor C. H. SCHULTZ, M.D. (*With Woodcuts.*) . 537

On the Influence of the Nerves on the Development of the Muscular System. By Professor ANTONIO ALESSANDRINI . . . . 543

### MEDICINE,

#### PATHOLOGICAL, PRACTICAL, AND THERAPEUTICAL.

On Pneumonia of the Old. By MM. HOURMANN and DECHAMBRE . . ib.

On Gangrene of the Lungs in the Insane. By J. GUISLAIN, M.D. . . 546

On the Nature and Mode of Action of Cantharides. By Dr. DOMENICO NARDO, of Venice, and Dr. TOMMASO PULLINO, of Alba . . . 548

On the Softening of the Mucous Membrane of the Intestinal Canal in Children. By Dr. DROSTE, of Osnaburg . . . . . . 551

On the Treatment of Tympanites with Camphor, Musk, and Ammoniacum. By Drs. TRADINI and SANTOLI . . . . . 552

An Enquiry into some Points of the History of Leucorrhœa. By Dr MARC L'ESPINE . . . . . . . 553

On the Use of Chloride of Soda in Intermittent Fevers. By Dr. GOUZEE . ib.

On the External Application of Croton Oil in Affections of the Larynx. By Dr. ROMBERG . . . . . . . 554

Expectoration of Bronchial Polypus, independent of Croup. By Prof. CASPER . ib.

On Incontinence of Urine. By M. MONDIERE . . . . 555

Formula for an artificial Chalybeate Water . . . . ib.

New Method of applying Cinnabar Fumigations . . . ib.

### SURGERY.

On Diseases of the Lymphatic System. By Professor VELPEAU . . 556

PAGE

On Vesico-Vaginal Fistulæ, and on a new Method of treating them. By M. Jobert . . . . . . . 561

On the Cure of Erectile Tumours. By Professor Lallemand, of Montpellier . 564

On certain Modifications in the Treatment of Hernia. By M. Gerdy . 566

On the Employment of Muriate of Barytes in the Treatment of White Swellings. By M. Lisfranc . . . . . . 567

On the Cure of Intestinal Fistulæ by the actual Cautery. By Dr. Fingerhuth . ib.

Case of Local Hereditary Relaxation of the Skin. By Dr. Graf, of Königsberg . 568

MIDWIFERY.

History of a successful Case of Cæsarean Section. By Dr. Meyer, of Minden . ib.

Case of Extra-uterine Pregnancy. By Dr. Löscher, of Lübben, and Professor Busch, of Berlin . . . . . . 570

Case of Fatal Hemorrhage from an Erectile Tumour of the Uterus. By Professor Kilian, of Bonn . . . . . . 571

HYGIÈNE.

On the Causes of Colica Pictonum amongst the Workmen who prepare White Lead. By M. Chevallier . . . . . 573

### Part Fourth.—Medical Intelligence.

On the Present State of Medicine in Prussia. By J. F. C. Hecker, m.d., Professor of Medicine in the University of Berlin.—Second Report: Medical Societies in Prussia . . . . . . . 577

On the Present State of Medicine in the United States. By Robley Dunglison, m.d., Professor of the Institutes of Medicine and Medical Jurisprudence in Jefferson College, Philadelphia.—Article I.: Of the Medical Institutions . . . . . . . 583

Report of the Medical Section of the British Association for the Advancement of Science. Held at Bristol, in August, 1836 . . . 594

Report of the Meeting of the Provincial Medical and Surgical Association . 601

Southern Branch of the Provincial Medical and Surgical Association . . 606

Report of the Court of Examiners to the Society of Apothecaries . . ib.

Medical Witnesses' Bill . . . . . . . 607

University of Edinburgh. Prizes to be given by the Medical Faculty . 609

Teachers of Anatomy and Surgery. To Gentlemen applying for the Recognition of the Royal College of Surgeons in London, as Teachers of Anatomy and Surgery . . . . . . . . 610

Botanical Society of Edinburgh . . . . . . 611

The New Registration Act . . . . . . ib.

OBITUARY.—Dr. Henry . . . . . 612

Professor Hufeland . . . . 613

List of Books received for Review . . . . . ib.

